# Practice organisational characteristics can impact on compliance with the BTS/SIGN asthma guideline: Qualitative comparative case study in primary care

**DOI:** 10.1186/1471-2296-9-32

**Published:** 2008-06-04

**Authors:** Sharon Wiener-Ogilvie, Guro Huby, Hilary Pinnock, John Gillies, Aziz Sheikh

**Affiliations:** 1 General Practice Unit, NHS Education Scotland, Edinburgh, UK; 2 Centre for Integrated Healthcare Research: School of Health in Social Science, University of Edinburgh, Edinburgh, UK; 3 Allergy & Respiratory Research Group, Division of Community Health Sciences: GP Section, University of Edinburgh, Edinburgh, UK; 4 Selkirk Health Centre, Scottish Borders, UK

## Abstract

**Background:**

Although the BTS-SIGN asthma guideline is one of the most well known and widely respected guidelines in the world, implementation in UK primary care remains patchy. Building on extensive earlier descriptive work, we sought to explore the way teamwork and inter-professional relationships impact on the implementation of the BTS-SIGN guideline on asthma in general practice.

**Methods:**

Qualitative comparative case study using nine in-depth interviews and 2 focus groups with general practitioners and practice nurses, involved in delivering asthma care. Participants were purposively recruited from practices in a Scottish health board with high and low compliance with the BTS-SIGN asthma guideline.

**Results:**

There was a marked difference in the way respondents from practices with high compliance and respondents from practices with low compliance spoke about the value of guidelines and the challenges of implementing them. On both accounts, the former were more positive than the latter and were able to be more specific about the strategies they used to overcome barriers to implementation. We explored the reason for this difference in response and identified practice organisation, centring on delegation of work to nurses, as a factor mediating the practice's level of compliance. Effective delegation was underpinned by organisation of asthma work among practice members who have the appropriate level of skills and knowledge, know and understand each others' work and responsibilities, communicate well among themselves and trust each others' skills. It was the combination of these factors which made for successful delegation and guideline implementation, not any one factor in isolation.

**Conclusion:**

In our sample of practices, teamwork and organisation of care within practices appeared to impact on guideline implementation and further larger studies are needed to explore this issue further. Isolated interventions such as measures to improve staff's knowledge or increased clinical resource and time, which are currently being considered, are unlikely to be effective unless practices are supported in developing their teams in a way which supports the deployment of these resources.

## Background

Worldwide, an estimated 300 million people suffer from asthma and 255,000 people died of asthma in 2005 [1]. Effective primary care management asthma is vital to minimise morbidity and mortality.

The British Guideline for the Management of Asthma [2] was published jointly by the Scottish Intercollegiate Network (SIGN) and the British Thoracic Society (BTS) in February 2003 and has since been updated annually with revisions placed upon both organisations' websites [3]. The key messages for primary care focused on: i) making an objective diagnosis of asthma; ii) employing stepwise pharmaceutical management; and iii) encouraging self-management education including provision of asthma action plans [4,5].

Guidelines have only a limited impact on practice [6]. While many approaches have been trialled to improve guideline implementation [7-13], evidence to support decisions about which guideline dissemination and implementation strategies are effective, under different circumstances, is limited [14].

Recognised barriers for guideline implementation include lack of awareness of the guideline, lack of agreement with, or belief in the guideline recommendations, lack of confidence, knowledge or skills to implement the guideline, lack of outcome expectancy, environmental related barriers such as insufficient resources, subjective norm and patient related barriers [15-19]. The literature is, however, relatively silent about what mediates these barriers and how to overcome them.

It is, perhaps, surprising that the role of inter-professional relationships and organisation of work within teams has not been extensively explored in relation to guideline implementation in primary care [14]. There is some evidence that good inter-professional collaboration is associated with better quality patient care [20,21], and there is a literature on the factors which create good inter-professional teamwork [22,23].

Our study of the primary care implementation of the three key recommendations of the BTS-SIGN guideline in a rural Scottish Health Board (the authority managing both acute and primary care) is unique in that it was carried out in three phases. The initial two phases (reported elsewhere [24])consisted of an audit of all general practices which sought to identify compliance with the recommendations, followed by a postal survey of general practitioners and practice nurses to identify perceived barriers and facilitators to implementation. Fifteen (63%) of the 24 practices in the Health Board participated in these two components of the study. The findings of the postal survey suggested that poor co-ordination of care and poor team work in general practices were barriers for the implementation of the recommendations. We now report the findings of the third, qualitative phase, in which we used the insights gleaned from the earlier work to explore in depth the way teamwork and inter-professional issues in teamwork relate to variation in implementation.

## Methods

Ethical approval for the study was granted by the participating Health Board's Research Ethics Committee.

### Design

Using a comparative case study design [25], we carried out individual in-depth semi-structured interviews with general practitioners and practice nurses involved in asthma care from five practices selected to represent variation across size and level of compliance with guideline recommendations. In addition, we convened focus groups with general practitioners and nurses, involved in asthma management, from two different primary care teams to test and explore how team members together discussed and debated the issues. The focus groups also included secondary care practitioners.

### Sampling strategy

#### Interviews

Based on the results of the audit of compliance with guideline recommendations and practice size, we used purposive sampling to identify practices for the study. Participating practices' were ranked with respect to their compliance with each of the three guidelines recommendations. We then calculated an average rank for each practice, assuming an equal weight for each recommendation. Finally, we developed a grid which showed practices' compliance with the recommendation against practice size. From this grid we selected five practices, which showed variation across both dimensions. We wanted to involve small, medium and large practices with a high and low level of compliance, i.e. six practices. We selected five as we were unable to identify a large practice with high compliance from our primary study. Table 1 provides a description of participating practices and clinicians.

**Table 1:** Characteristics of participating practices

**Practice 1: small practice with high compliance**
• **Interviewee**: One female GP. No PN in practice
• **Size: **A small dispensing practice (list size < 3000 patients)
• **Compliance: **The best audited compliance with the three key recommendations of the BTS-SIGN guideline in the study. i.e. always used objective testing and add on therapy as indicated by the guideline with 25% of patient reporting to have Asthma action plan.
• **Staff**: GPs < 4, no practice nurse, part time pharmacist on site.
**Practice 2: Small Practice with low compliance**
• **Interviewees: **One male GP and PN
• **Size**: A small dispensing practice (list size < 3000 patients)
• **Compliance**: Poor audited compliance with the three key recommendations of the BTS-SIGN guideline for primary care. i.e 75% of new asthmatic had objective testing confirming diagnosis, 68% of patients receiving 800 mcg inhaled corticosteroids daily were on appropriate add on therapy but there was no provision of asthma action plans.
• **Staff**: GPs < 4, practice nurse (with no asthma diploma, or prescribing ability), no pharmacist.

**Practice 3: Medium practice with high compliance**
• **Interviewees**: one male GP and one PN
• **Size**: A Medium practice (list size 3,000–8,000 patients)
• **Compliance**: Good audited compliance with the three key recommendations of the BTS-SIGN guideline for primary care i.e. all newly diagnosed patients had objective testing confirming diagnosis, 55% of patients receiving 800 mcg inhaled corticosteroids were on appropriate add on therapy and 52% of patients we surveyed had asthma action plans.
• **Staff**: GPs > 3, asthma nurse with prescribing abilities and asthma diploma.

**Practice 4: medium practice with low compliance**
• **Interviewees**: One male GP and one PN.
• **Size**: A medium practice (list size 3,000–8,000 patients)
• **Compliance**: Poor audited compliance with the three key recommendations of the BTS-SIGN guideline for primary care i.e. 33% of new asthmatic had objective testing confirming diagnosis, 50% of patients receiving 800 mcg inhaled corticosteroids daily were on appropriate add on therapy and only 15% of patients reported to have asthma action plans.
• **Staff**: GPs > 3 with a non prescribing PN with asthma diploma.

**Practice 5: Large practice with low compliance**
• **Interviewees**: one male GP and one PN.
• **Size**: A large practice (list size > 8000 patients)
• **Compliance: **Poor audited compliance with the three key recommendations of the BTS-SIGN guideline for primary care. i.e. 23% of new asthmatic had objective testing confirming diagnosis, 72% of patients receiving 800 mcg inhaled corticosteroids daily were on appropriate add on therapy and only 12% of patients reported to have asthma action plans.
• **Staff**: GPs > 6 and 2 asthma PNs without prescribing abilities but with asthma diplomas.

#### Focus groups

We conducted practice based focus groups with two medium size practices with moderate compliance.

### Data collection

Interviews and focus groups were conducted by the project researcher (SWO) and took place at the practices at a time that suited clinicians. Data collection took place between May and July 2006.

#### Interviews

The semi-structured interview questions were developed by the multidisciplinary research team, drawing on published literature and responses from our postal survey [24]. The interviews sought to gauge the opinion and experience practitioners had with the use of objective testing in the diagnosis of asthma, stepwise management and asthma action plans, focussing on what facilitated and hindered their implementation. Current work arrangements, communication methods and relationships in the team (especially between doctors and nurses), delegation of responsibilities in relation to asthma management and patients' related issues were explored. Each interview lasted approximately 45 minutes. The topic guide for the interviews is available as appendix 1.

#### Focus groups

We used two case scenarios, developed by the research team, to facilitate discussion amongst clinicians and test our emerging finding that team organisation and communication had an impact on guideline implementation (Additional file 1). The first facilitated the identification of barriers for the use of objective testing in the diagnosis of asthma. The second focused on the use of asthma action plans and facilitated a discussion around their use. Focus groups lasted for approximately 45 minutes.

### Data analysis

Interviews and focus groups were taped, transcribed and checked for accuracy. Three members of the research team each read three transcriptions and together agreed a coding frame. Further sub-themes were developed using NVivo software.

We grouped the codes together in broad themes as follows: a) adapting to changes in general practice, managing time and prioritising work; b) level of agreement with the guidelines; c) knowledge and skills in guideline implementation among GPs and nurses; d) perceived patient issues; and e) practice organisation. While themes a, b, c and d were 'in vivo' codes suggested by respondents' own language, theme e, practice organisation, was of a higher analytical abstraction, constructed from respondents' descriptions of the way their practices were organised. It contained the codes organisation of asthma work, including distribution of work and responsibilities among team members, delegation of tasks, hierarchy in decision-making, trust and communication.

We then explored whether themes we had identified related to each other and to the level of compliance with the guidelines in our respondents' practices. Overall, we were satisfied that data saturation was achieved with our sample in relation to the main study objective.

## Results

Altogether four nurses and five GPs were interviewed. All clinicians we approached agreed to be interviewed. Two focus groups were conducted. The first (focus group 1) included four principal GPs, a trainee GP, practice nurse and a secondary care specialist respiratory nurse. The second focus group (focus group 2) included two principal GPs and a practice nurse.

Below, we present data illustrating the main themes identified. The presentation also aims to convey how our deepening engagement with the data led us to identify practice organisation as a key factor mediating guideline compliance. The difference in response from respondents from practices with high levels of compliance on the one hand, and practices with low levels of compliance on the other, was a striking early finding which made us look closer at practice organisation and we present data excerpts in a way which illustrates this difference.

### 1. Dealing with change, managing time and the need to prioritise workload

The constant need of general practice to adjust to external changes emerged as a contextual factor impacting on guideline implementation. Changing old habits and adjusting to new concepts was highlighted as difficult in an environment of constant change, where the task of updating knowledge and actually implementing new developments increases workloads.

Small practice with high compliance (practice 1):

GP: "... *I don't know, I don't want to get too defensive but we are bombarded with change all the time and it's very hard to keep up to date, especially someone like myself who is over 50. We try hard to keep up to date but a lot of things have changed and there a lot of inhalers, in my time, come out ... so we're learning on the job...but it's that knowledge to action power them, you can know a thing and not know it for quite a long time before you do it."*

Both GPs and nurses felt that lack of time was a barrier for the implementation of the recommendations. A large patient agenda within a short appointment meant that carrying out objective testing, or providing an asthma action plan was not always a priority. Clinicians also identified lack of time for practice meetings and discussions.

Respondents from highly compliant practices were more positive about challenges and how to tackle these than respondents from practices with low compliance. They were also more specific about the way those issues impacted in different situations, whereas respondents from practices with low compliance tended to speak in more general and negative terms.

Small practice with high compliance (practice 1)

GP in relation to lack of time and asthma action plans:

"... so there is not quite the emphasis at the annual review if everything is stable to suddenly find this bit of paper (asthma action plan) and fill it in. It seems a bit contrived and I am sure patients feel that it's a bit hypothetical as well and you're pushed for time and, let's face it, doesn't happen."

Small practice with low compliance (practice 2):

GP: *In relation to asthma action plans: "... it seems to be a good idea but there seems to have been so many other things happening that we never really got around to doing it. I think in the pile over, there is a document about management plans but I never really got anywhere with it."*

### 2. Agreement with the guideline

Within this context of constant change and the lack of time to adapt, the level of confidence in guidelines emerged as an issue. We observed that the language used to describe this issue differed between practices with high and low compliance. Clinicians from practices with high compliance described guidelines positively and emphasised the importance of implementing recommendations:

Medium sized practice with high compliance (practice 3):

PN: "*I have been introducing salmeterol in quite a lot of people obviously with the new guideline."*

In contrast, respondents from practices with low compliance were more sceptical about aspects of the BTS-SIGN guideline, sometimes questioning the recommendations:

Large practice with low compliance (practice 5):

PN: *objective testing. " I think it has its use but it's limited and you've got to assess the patient and whether or not it's viable...... we know there was an update SIGN guideline in November...there was talk about not doubling up 'preventers'... but we were not been able to find the evidence behind this."*

### 3. Perceived patients' issues

The contrast in language between respondents from high and low compliant practices was particularly striking when they discussed the way patient behaviour impacted on guideline implementation.

Respondents from practices with high compliance tended to relate to the patients' perspective:

Medium practice with high compliance (practice 3):

PN: *" you always get people who will comply better than others and that's just a general thing with anything. The job is not to tell them what to do, but to advise them of the outcome of not complying the way they should. You can also try and give them the knowledge to try and encourage them."*

Small practice with high compliance (practice 1)

GP: " sometimes I think we're not clear enough about what we're wanting so there's a communication problem, we are not explaining carefully enough what we want and why we want it."

Respondents from low compliant practices were more likely to point out patients' lack of motivation, responsibility for their own disease management and non-attendance for reviews as a barrier for guideline implementation. These respondents also perceived lack of confidence and fear among patients associated with misunderstanding and confusion about self management.

Large practice with low compliance (practice 5):

GP:*"... literally I had somebody beyond the joke ..I said "What do you think?" and he looked at me goggle eyed and he said "you are the F' ing doctor". I had to laugh and I thought '"Oh my God". Its horses for courses and generally speaking our patients tend to be a bit more" just tell me what to do, doctor."*

PN: " [Patients]*not all are confident to increase inhalers on their own, however much evidence or encouragement you give them, they want to come in and be told. They are not confident at recognising the signs or the symptoms ...Also have to look at their ability to increase and do they have the intellect to remember to reduce them,"*

Responses suggested that the way practices organised their contact with patients influenced patient behaviour and shaped the practice-patient relationship:

For example, the medium practice with high compliance (practice 3) adopted a relatively 'dictatorial' approach:

GP: "From the administrative point of view, I work in conjunction with one member of clerical staff and the practice nurse and about 3 or 4 times a year we will have a sit down .... reaching those patient's who haven't actually turned up for asthma review. The practice nurse and the member of staff, the administrative staff meet regularly, and the administrative member of staff is responsible for sending out the three letters. The first letter is a general letter, the second letter is a slightly more persuasive letter and the third letter which is signed by me says that if they don't turn up for the review they will not get their medication, or there is a chance that they won't get their medication because we are dealing with expensive products here and potentially fatal illnesses and so normally the third letter gets a response from most people. Last year I probably stopped about three people's medication and as far as I am aware they have not turned up for it, so they probably don't need it."

By contrast, the small practice with high compliance (practice 1), sought advice from colleagues about how they could 'get through' to a poorly compliant patient:

GP: "..we all struggled with(the patient) and I have seen him and done some blood test etc and I have discussed it with the other GPs and they made some suggestions about how we could try and get through to him.. so maybe just discuss who would be best., who knew them best, who felt they had the best communication and just follow up by phone."

### 4. Knowledge and skills in the primary care team: issues of delegation of work to nurses

Practice organisation also appeared to be a factor when we explored the knowledge and skills needed for effective guideline implementation. Delegation of routine chronic disease management, including asthma care, to nurses is standard practice and the knowledge and skills of nurses (including ability to prescribe) was perceived to be important by all clinicians.

Medium sized practice with high compliance (practice 3):

GP: "the practical clinical point of view: the management of asthma as a chronic disease is done almost in it's entirety by the practice nurse who has her necessary diplomas but she is also a prescriber ...and she is given the necessary support by myself or through myself by the practice from the point of view of administration."

It was not just a matter of individual clinicians' knowledge, however, but of the distribution of skills and knowledge across the team. GPs skills and knowledge also impact on nurses' work:

Large practice with low compliance (practice 5):

PN: *"It happens a lot, they (the patient) come in for an acute attack, the GP instantly increases their inhalers and 9 out of 10 I am trying to get them (the GPs) to look at compliance and probably the reason for the patient having an acute attack is because they haven't been taking their inhalers."*

The relationship between the nurse and the rest of the practice was a balance between specialisation and delegation on the one hand, and sufficient general knowledge retained by all team members, on the other. For example, delegation of responsibilities between GPs and nurses could mean that GPs had little understanding of the use of asthma action plans effectively marginalising the concept.

Large practice with low compliance (practice 5):

GP: *" When you ask personally how do I feel (about asthma action plans), I suppose I feel personally shaky because I don't have the working facility with providing patients with written plan... it is normally the practice nurses that are involved with them...that's one thing I have noticed with the bigger practice and with the increasing specialisation within primary care, in particular the chronic disease management field, is that you find yourself getting a little bit de-skilled or marginalised with regards to things like self management plans."*

There were clear differences between the ways in which delegation to practice nurses was described by respondents from high and low compliant practices. Attitudes to delegation reflected both to personality, experience and training of individuals, and processes in particular practices:

Medium practice with high compliance (practice 3):

GP: *"I have no problem at all with delegating that at all so long as you know what your delegating, who you're delegating to and that you're giving them sufficient support from the point of educational updates etc etc so.. I've worked with N for years, I know what she's like and she's absolutely utterly dependable and so I don't have a problem with that but that's perhaps more a reflection of my personality because a lot of us in the practice delegate to the appropriate people otherwise we just couldn't get on with our job."*

Medium practice with low compliance (practice 4):

PN: *" If they (GPs) gave a patient a prescription for inhalers etc and I didn't agree with it I wouldn't say, as I said before, I would maybe speak to them (the GPs) and put my point of view forward and if they (the GPs) agreed with me I would maybe change it, but I would not say, I wouldn't be forceful enough to say "that's not right". I would have a discussion, but no I don't think so because ultimately I don't sign prescriptions, I've not done the nurse prescribing and ultimately they sign the prescription so they have the last say, but 9 times out of 10 we come to an agreement."*

Ways in which practice communication and team relationship shaped delegation of routine asthma work to nurses was also suggested in focus group discussions:

GP from focus group 1:

"*We just went over the local hypertension guidelines recently ... and we discussed them and everyone has different ideas and I think that the thing that is key, sitting down and saying, and also if you sit down and say this is what we're going to do then if R (the practice nurse) comes across to me and says well actually G (Dr) you're not doing it then if you've agreed it, I think a lot of time with guidelines it is about agreeing that in the practice you're going to do them."*

### 5. Organisational issues

Delegation of work to nurses might be a lynchpin of effective guideline implementation and we were becoming aware of practice organisation as a key factor shaping the way delegation of work to nurses was managed. We turned to our data on practice organisation to explore this further. Organisational differences between our selected practices are described in Table 2.

**Table 2:** Practice organisation and level of guideline compliance

**Small practice with high compliance**	• **Organisation of asthma management **GPs were aware of how the other GPs worked, with all partners working in a similar way and using the pharmacist in a similar way.
	• **Delegation**: No delegation of work to other professions. All GPs were dealing with both acute and chronic management of asthma.
	• **Hierarchy: **No hierarchy in relation to asthma management as no GP lead on asthma, with other professional such as pharmacist being highly appraised and considered as part of the team. GPs often made decision together.
	• **Trust and confidence **in all partners ability to manage patients
	• **Communication and team members' access to each other**: informal, but coffee time provided a set time for communication. GPs tried to involve other professionals in educational meeting they held.
**Small practice with low compliance**	• **Organisation of asthma management**: no consistent approach for the roles and responsibilities of the GPs and the nurse in relation to asthma management. No practice asthma protocols. Lack of awareness of how other team members or systems in the practice worked.
	• **Delegation**: partial and inconsistent delegation of responsibilities between GPs and nurse.
	• **Hierarchy**: There were clear hierarchical relationship between GPs and the nurse
	• **Trust and confidence**: GP cautious about nurse management of patients.
	• **Communication and team members' access to each other **informal, unstructured and with no set times

**Medium practice with high compliance**	• **Organisation of asthma management**: local asthma protocols, asthma team which included the practice nurse, lead asthma GP and administrative support which met regularly and ensured a systematic approach for patients' annual reviews, while ensuring flexibility of appointment for patients. Consistent approach for both the diagnosis and management of asthma as those functions were carried out by PN. Practice members of all levels worked closely with one another.
	• **Delegation**: The responsibility for asthma diagnosis and management was exclusively delegated to the PN. Nurse changed medication and agreed action plan with the patient.
	• **Hierarchy**: no hierarchy between GPs and nurse in relation to asthma management
	• **Trust and confidence**: GPs were happy to delegate responsibilities and trusted the nurse to manage patients. Nurse was confident in her ability to influence the GPs and change the way they work
	• **Communication and team members' access to each other **communication within the practice was reported to be good and take both informal and formal forms with regular meetings of asthma team and weekly meetings of the GPs which nurse can attend.

**Medium practice with low compliance**	• **Organisation of work**: no practice asthma protocols. No consistent approach for the diagnosis of asthma or stepwise management of asthma among the GPs. The practice did not actively check patients' compliance with medications but offered flexible appointment system. Lack of administrative support to nurse. Lack of awareness of how others in the practice worked.
	• **Delegation: **GPs responsible for the diagnosis of asthma and treatment of acute attacks and the PN annually reviewed patients.
	• **Hierarchy**: hierarchical relationship between GPs and nurse. GPs made the decisions
	• **Trust and confidence**: nurse lacking confidence to challenge GPs if not agreeing with their decisions.
	• **Communication and team members' access to each other: **no formal meetings, but a lot of informal communication and practice nurse was comfortable with current informal discussions with lead GP for asthma. Practice meetings did not include the nurse and nurse felt that there was no time for multidisciplinary meetings.

**Large practice with low compliance**	• **Organisation: of work: **lead asthma GP for in-house chronic disease management structure with agreed asthma protocols, regular asthma team meetings. Systems for reviewing unstable patients and flexible appointments. Inconsistent approach to diagnosis and review of compliance by GPs and lack of awareness of how nurses worked.
	• **Delegation**: GPs diagnosed and treated acute attacks and nurses reviewed patients and looked at compliance and education.
	• **Hierarchy**: a degree of hierarchical relationship between GPs and nurses in the practice.
	• **Trust and confidence**: nurses felt trusted by the GPs but had difficult influencing the GPs ways of working.
	• **Communication and team members' access to each other **nurses felt they could discuss any problem with the lead GP and there were set asthma team meeting and practice based chronic disease management meetings on a regular basis. There was a rigid arrangement for communication with GPs: daily set (formal times) in the practice where GPs were available to discuss any issues with other health professionals.

Effective delegation of work depended on a range of features being in place.

For example, in the medium practice with high compliance, the GP expressed trust and confident in the practice nurse as she was well qualified (had an asthma diploma and was a nurse prescriber). The nurse was viewed as a confident and effective communicator, something which empowered her to influence GPs' behaviour. This facilitated a 'flat' hierarchy in the practice, which, in turn enabled the delegation of responsibilities in it's entirely to the nurse with the required additional educational and administrative support to help her, resulting in a consistent approach to asthma diagnosis and management.

In the small practice with high compliance, there was no nurse and the practice had a flat organisation with good channels of communication among GPs. GPs shared decision-making and developed a consistent approach to asthma management.

As the nurse in the small practice with low compliance had limited asthma knowledge and skills (no formal qualification although had experience), the GP was less trusting in her abilities and therefore was less keen on devolving entire responsibility to her. There was patchy communication within the practice. The combination of these factors resulted in an inconsistent approach to asthma care.

In the medium practice and large practices with low compliance, there was partial delegation of responsibilities to the nurses. Again, this had several interconnected reasons. Both practices displayed a more hierarchical approach to asthma management where the GP was still seen as the lead clinician, despite being less involved in many aspects of their care (encouraging compliance, self management education). The nurses' ability to influence the behaviour of the GPs was reduced despite being qualified (both had an asthma diploma although were not prescribers).

In summary, practices with low compliance had less consistent approach to diagnosis and management and less awareness about how others in the practice worked. GPs and administrative staff did not routinely communicate relevant information (for example about an asthma admission) to the nurses as the GP was seen as the lead for patient management. It is worth noting, that these 'softer' organisational features could negate otherwise good systems of protocol-driven care, as was the case in the large practice with low compliance. In contrast, practices with high compliance had in place effective channels of communication, skills and knowledge were distributed among practice members and there was mutual respect and trust.

## Discussion

Organisation of care, communication and decision-making within practices appear to have a major impact on guideline implementation. Dealing with change, managing time, the need to prioritise work and patient behaviour were issues concerning all practices in terms of guideline implementation. However respondents from highly compliant practices were more positive about these challenges and tended to describe a more positive attitude to guideline recommendations.

Highly compliant practices were also more sympathetic to the patient's perspective. A consistent approach to diagnosis and management of asthma, defined roles and responsibilities of doctors and nurses within the practice, good communication between team members, trust and confidence between doctors and nurses and flatter hierarchical arrangements all appeared to support guideline implementation.

### Strengths and limitation of this work

The sample for this study is small but follows the recommended approach of sampling according to a theoretically-based framework. Examination of more practices or clinical teams in other geographical and clinical areas will test transferability and allow theory building around the importance of team work in guideline implementation. The multidisciplinary research team, comprising clinicians, GP academics, a social anthropologist and a health services researcher, ensured that multiple perspectives and knowledge were brought to bear on the analysis. This too, increased the rigour of the analysis.

### Interpretation of findings within the broader guideline implementation literature

From the beginning of this study we were intrigued to find that respondents from high and low complaint practices spoke in very different ways about workload and rate of change, trust in the guideline and patient behaviour as issues in guideline implementation. Our data imply that highly compliant practices may also relate to their patients differently than practices with poorer compliance. They understand, and are able to respond to, practical issues patients face in becoming more involved in their own asthma care. The organisation of clinical work, communication and decision-making within the team emerged as a key underlying factor mediating these responses and their relationship to levels of compliance with the guidelines. For example, managing change and increased workloads is easier in well-organised teams with open communication and high levels of trust. This would account for our finding that respondents from highly compliant practices spoke about the challenges of change and workload in more positive terms than respondents from practices with low compliance, and were also more specific about what these challenges entailed and how they addressed them to effect implementation of the guidelines.

Our findings support the suggestions by Checkland et al [26] that barriers to guideline implementation are constructed by clinicians, based on their values and beliefs, and therefore have to be viewed in their organisational context taking into account underlying social relationship within practices.

As we engaged with the data we found that practice organisation, centring on delegation of work to nurses, was a factor mediating the practice's level of compliance. Delegation needs to be underpinned by distribution of responsibility amongst practice members who have the appropriate level of skills and knowledge to be able to share the workload, know and understand each others' work and responsibilities, communicate well among themselves and trust each others' skills. It was the combination of these factors which makes for successful delegation and hence guideline implementation, not any one factor in isolation.

Our study echoes findings by others in relation to barriers to guideline implementation [15,19], such as capacity and resources, disagreement with the guideline, knowledge and skills of staff and patient's issues but adds the dimension of organisation of primary care teams, in particular as it affects relationships between doctors and nurses, as a hitherto unexplored dimension in successful guideline implementation. Our findings are supported by Vinas and Castel [22] and Shaw et al [23], that who suggest that good team working was enhanced by common defined goals, adequate communication and participations of all team components in decision making. Few team meetings and lack of team work training and hierarchical structures hindered team work for our practices with low compliance.

In the UK, the Quality and Outcome Framework (QOF) of the General Medical Services contract rewards practices that achieve targets in, for example, providing regular asthma reviews. All practices in our study achieved maximum level of QOF points, but their organisational approach to care delivery and the recording of delivery, (for example, the strategies they used to deal with the challenge of non-attenders) suggest that the way practices use and manage resources to achieve these targets vary. This may have an impact on the patient's experience of the care they receive.

## Conclusion

Our findings suggest implications both for further research and practical measures to improve guideline implementation. Further research needs to challenge and refine our findings through comparative work in other settings to allow theory building around the relationship between team work and guideline implementation.

Our study suggests that isolated interventions such as measures to improve staff's knowledge or increased clinical resource and time, which are currently being considered, are unlikely to be effective unless practices are supported in developing their team in a way which supports the deployment of these resources.

As a range of organisational factors may influence effective guideline implementation, support for practices should address the interaction of these factors, rather than individual factors in isolation. This will have important implications in the UK, where under the terms of their General Medical Services contract, general practices are rewarded for achieving evidence based targets.

## List of abbreviations used

BTS-SIGN – British Thoracic Society – Scottish Intercollegiate Guidelines Network. GP – General Practitioner. PN – Practice Nurse.

## Competing interests

AS co-chairs the British Thoracic Society's Science & Research Committee and HP chairs the BTS-SIGN asthma guidelines implementation group.

## Authors' contributions

SWO helped to plan the research, collected all data, carried out the analysis and wrote the paper. GH helped in the planning, design, analysis of the research and wrote the paper. HP helped in the planning and design of the research and the writing of the paper. AS had the idea for the research and took part in the discussion around the analysis and helped to write the paper. JG helped in the planning and the design of the research. All authors read and approved the final manuscript.

## Appendix 1: Interview schedule

1. Objective testing: what is your experience of using objective testing in the diagnosis of asthma-how do you go about it? (peak flow variability/spirometry).

- Do you ever give pick flow diaries...

- How quickly see nurse etc.

- How do you feel about the use of objective testing in the diagnosis of asthma?

Do you feel it is important to use objective testing?

- What factors encourage the use of objective diagnostic testing in your practice?

- Are there any factors that make it difficult to carry objective diagnostic tests on new patients in your practice?

- *Do you feel all partners work in the same way?*

- *Do you have protocols for the diagnosis of asthma?*

- *Do you feel everyone is following them in the same way?*

- *Do you feel the work is well co-ordinated?*

- *Do you have enough staff/clinics/is staff trained*

-

2. SMP (self management plans).

- How do you feel about the use of written SMP in the management of patients with asthma?

- How do you feel about providing patients with SMP yourself?

- In this study we found that SMP are not widely used in Borders why do you think that is?

- What can help to make the use of SMP more wide spread?

- What do you think self management of patients with asthma means?

- In what ways have you provided this in the past?

- Do you see it as something that needs to be done every so often or a continuous ...

- Whose role it ?

4. In the questionnaire, we have sent many GPs mentioned "team work" in relation to the implementation of the recommendations in general practice. How do you feel " team work" function in relation to asthma management in your practice?

If they say well: ask them what that means

• *In your opinion what are the characteristics of a team that "works well."*

• *What factors can influence team work within your practice (I am thinking personalities, environmental factors, resources etc)*.

5. How do you reach decision in your practice in relation to the management of "difficult asthmatic patients"?

6. What methods or type of communication do you use within the practice, to co-ordinate the care of patients with asthma (regular meetings, ad hoc discussions, case studies, review of protocols etc.).

7. From the questionnaire, it appears that delegation of responsibilities between GPs and nurses impacts on the implementation of the recommendation, how does it work in your practice?

-Do you feel GPs and nurses have different roles in the management of patients with asthma? (areas doctors good at/areas nurses good at)

• *If difference, in what way the differences impact on the implementation of there recommendation (the use objective diagnosis, ICS, and use of SMP*.

• *How can the work of GPs and nurses can be better co-ordinated in the management of patients with asthma?*

8. In the questionnaire, some of the GPs mentioned (that the patients themselves/or patients' behaviour impacts on the implementation of the recommendations, can you tell me more about this?:

• How can patients be motivated to, attend clinics.

• What can improve patients' compliance with SMP?

• Time limitation and individual consideration, how do you go about squaring the circle?

9. Tell me about your experience with recommendation 2.

Prompt: GPs often prescribe above 800 mcg/daily of ICS (say 4 puffs of 250 mcg daily, without initiating a trial of add on therapy. Why do you think this is happening?

Is cost an issue?

Are there any other factors?

• What can encourage GPs to use Add on therapy before increasing the dose of ICS above 800 mcg daily.

10. How well is the care of patients co-ordinated between primary and secondary care?

## Pre-publication history

The pre-publication history for this paper can be accessed here:



## Supplementary Material

Additional file 1 Case studies used in the discussion with the focus groups. Description of the case studies used in the discussion with the focus groups. Click here for file
