# Supplementary figures and images for: Practice organisational characteristics can impact on compliance with the BTS/SIGN asthma guideline: Qualitative comparative case study in primary care
Source: BMC Fam Pract. 2008 Jun 4;9:32. doi: 10.1186/1471-2296-9-32 (PMC2427031; doi:10.1186/1471-2296-9-32)

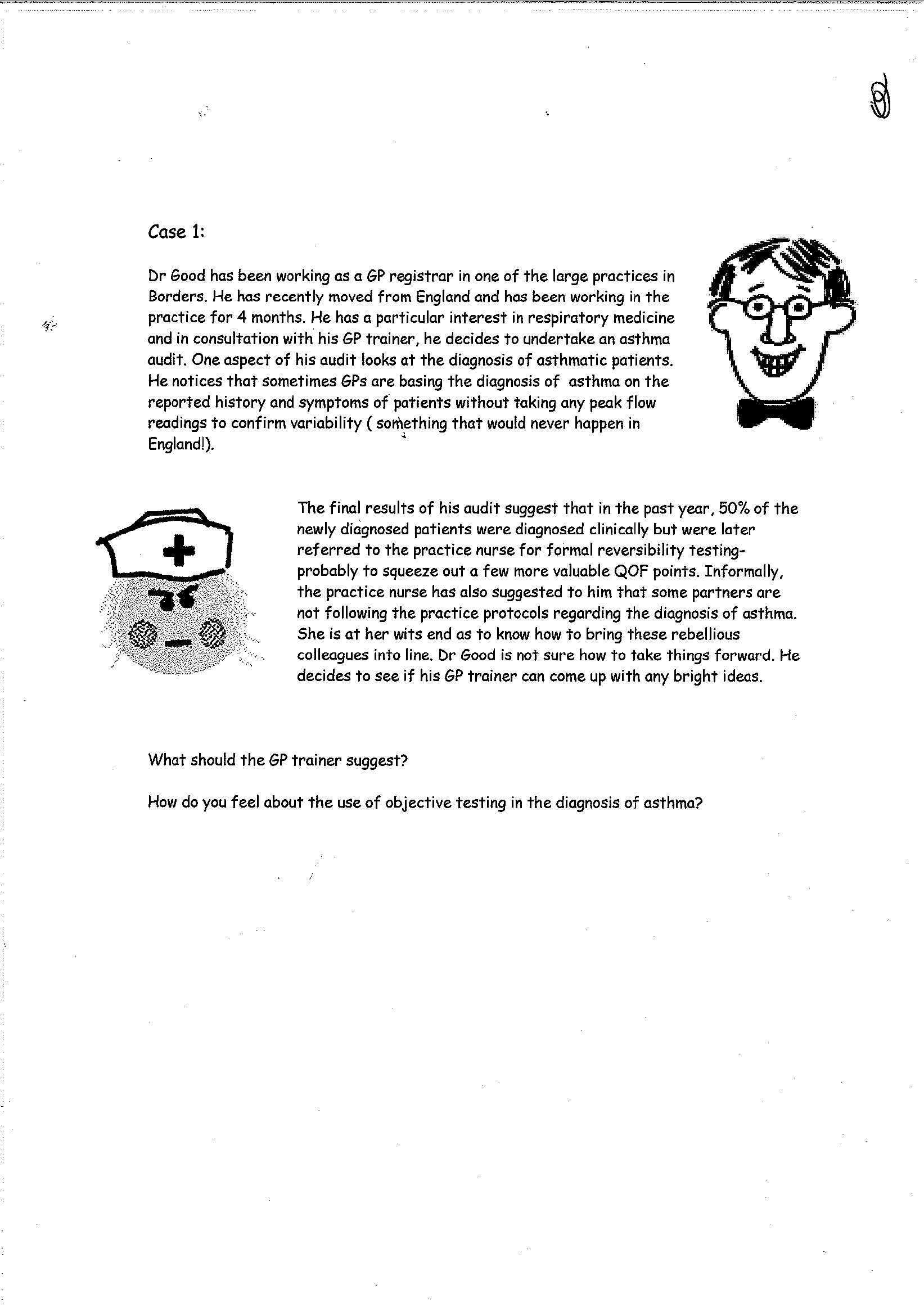
**Additional file 1**


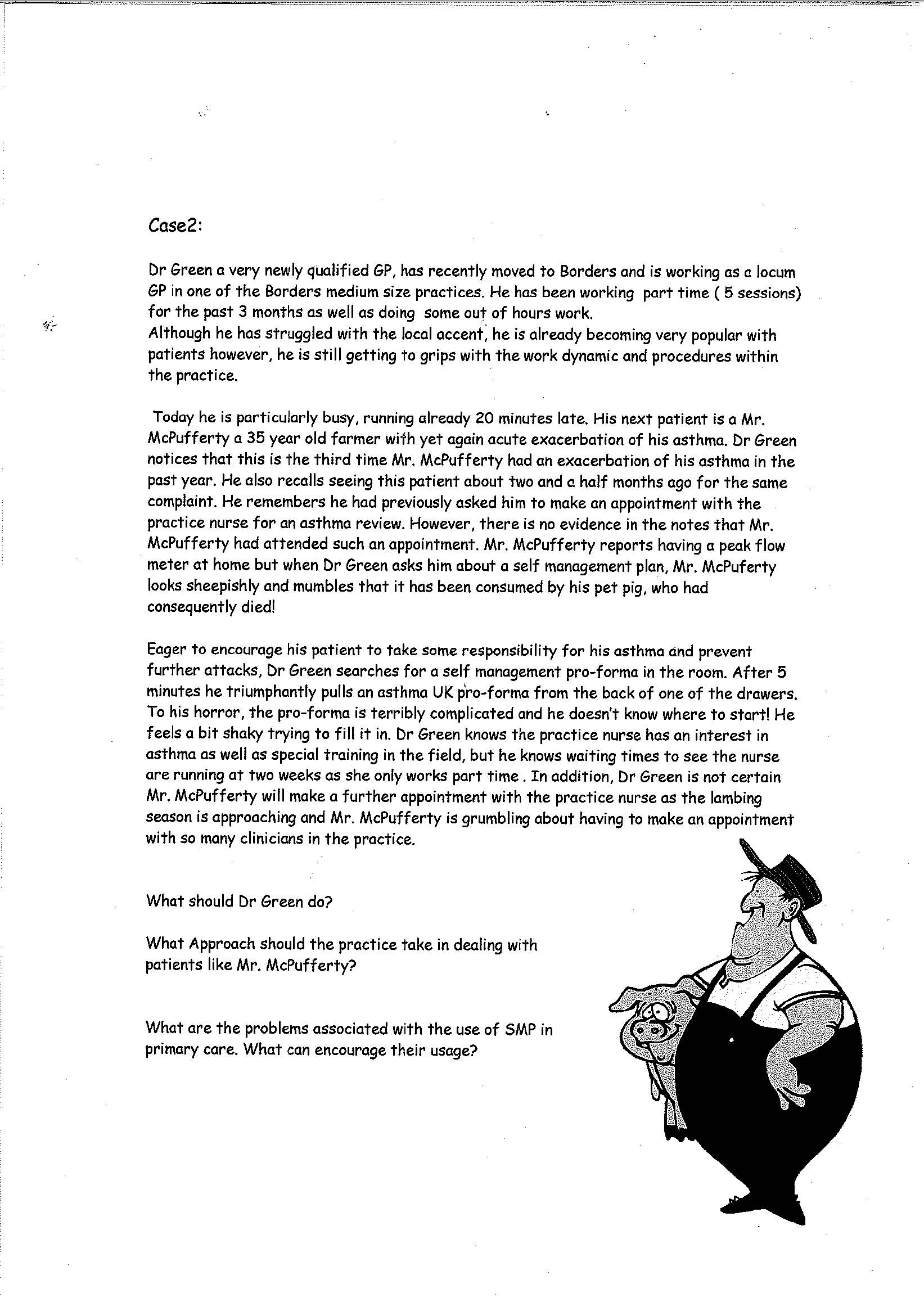

Supplement: Additional file 1 — Case studies used in the discussion with the focus groups. Description of the case studies used in the discussion with the focus groups. [file 1471-2296-9-32-S1.doc]
